# Gadolinium-Enhanced Extracranial MRA Prior to Mechanical Thrombectomy Is Not Associated With an Improved Procedure Speed

**DOI:** 10.3389/fneur.2018.01171

**Published:** 2019-01-09

**Authors:** Adrien Guenego, Naim Khoury, Raphaël Blanc, Mikael Mazighi, Stanislas Smajda, Hocine Redjem, Gabriele Ciccio, Jean-Philippe Desilles, Simon Escalard, Kevin Zuber, Pauline Chamard, Mylène Hamdani, Nahida Brikci-Nigassa, Malek Ben Maacha, Michel Piotin, Robert Fahed

**Affiliations:** ^1^Interventional Neuroradiology Department, Fondation Rothschild Hospital, Paris, France; ^2^HSHS Neuroscience Center, HSHS St. John's Hospital, Springfield, IL, United States; ^3^Biostatistics, Fondation Rothschild Hospital, Paris, France

**Keywords:** stroke, thrombectomy, magnetic resonance angiography, angiogram, angiography, magnetic resonance imaging, revascularization

## Abstract

**Objectives:** To assess whether performing a pre-intervention gadolinium-enhanced extracranial magnetic resonance angiogram (MRA) in addition to intracranial vascular imaging is associated with improved thrombectomy time metrics.

**Methods:** Consecutive patients treated by MT at a large comprehensive stroke center between January 2012 and December 2017 who were screened using pre-intervention MRI were included. Patients characteristics and procedural data were collected. Univariate and multivariate analysis were performed to compare MT speed, efficacy, complications, and clinical outcomes between patients with and without pre-intervention gadolinium-enhanced extracranial MRA.

**Results:** A total of 912 patients were treated within the study period, including 288 (31.6%) patients with and 624 (68.4%) patients without extracranial MRA. Multivariate analysis showed no significant difference between groups in groin puncture to clot contact time (RR = 0.93 [0.85–1.02], *p* = 0.14) or to recanalization time (RR = 0.92 [0.83–1.03], *p* = 0.15), rates of successful recanalization (defined as a mTICI 2b or 3, RR = 0.93 [0.62–1.42], *p* = 0.74), procedural complications (RR = 0.81 [0.51–1.27], *p* = 0.36), and good clinical outcome (defined by a mRS ≤ 2 at 3 months follow-up, RR = 1.05 [0.73–1.52], *p* = 0.79).

**Conclusion:** Performing a pre-intervention gadolinium-enhanced extracranial MRA in addition to non-contrast intracranial MRA at stroke onset does not seem to be associated with a delay or shortening of procedure times.

## Introduction

Recent guidelines of the American Heart Association (AHA) recommend performing emergency intracranial vessel imaging to demonstrate the presence of a large vessel occlusion (LVO) in mechanical thrombectomy (MT) candidates (Class I evidence) and suggest performing additional pre-intervention non-invasive extracranial vascular imaging (Class IIb evidence) (1). Obtaining extracranial vessel imaging is inherently implied when obtaining intracranial vessel imaging at centers that utilize computed tomography (CT) and CT angiography (CTA) as the primary screening tests for acute neurovascular emergencies. The super-fast acquisition speed of high quality angiographic imaging for both head and neck regions (intra- and extra-cranial), the single dose of iodinated contrast required for a comprehensive examination, and the low incidence of renal complications following CTA in stroke patients (2) are three pillars of CTA that don't lend themselves to magnetic resonance imaging (MRI) when employed for stroke acute screening. MRI screening consists of four short sequences, namely the diffusion-weighted imaging (DWI) (3), the fluid-attenuation-inversion-recovery (FLAIR) (4, 5), gradient-echo (GRE) or susceptibility-weighted-imaging (6, 7), and intracranial magnetic resonance angiogram (MRA) with time-of-flight (TOF) (1). These are sufficient for the acute medical or interventional management of ischemic stroke and for the diagnosis of intracranial hemorrhage. Acute intravenous thrombolysis decision may be based on data obtained from the above MRI sequences and intracranial LVO can be demonstrated using such MRI protocol. Imaging of extracranial vessels using MRI increases the patient screening time in this time-sensitive diagnosis (8) and requires the use of gadolinium contrast [which may be associated with renal (9) and cerebral (10) toxicity]. Although the AHA guidelines suggest that extracranial vessel imaging may “provide useful information on patient eligibility and endovascular procedural planning,” (1) this suggestion has not been scientifically demonstrated yet. We aimed to assess whether performing a pre-intervention extracranial vessels Gadolinium-enhanced MRA is associated with improved procedure speed.

## Methods

This is a single center retrospective analysis of prospectively collected data. The study protocol was approved by the institutional review board and informed consent was waived according to French regulations where the study was conducted.

### Population

We identified consecutive AIS patients who underwent MR based screening for acute neurovascular evaluation and who underwent MT at one comprehensive stroke center between January 2012 and December 2017. Included patients either presented directly to the study center where imaging screening and intervention occurred, or were accepted for interventional management at the study center after having received MRI screening at an affiliated referral center. No MRI imaging was repeated at the study center for referred patients. Patients characteristics (including initial NIHSS, occlusion site), MT procedural data (including rate of successful recanalization [i.e., TICI ≥ 2B], time from groin puncture to clot contact and to recanalization (minutes), time from onset to recanalization, complications [embolic or hemorrhagic]), and clinical outcomes (modified Rankin Scale [mRS] at 3 months assessed by certified neurologists) were collected. All included patients presented with symptoms of AIS and underwent a standardized stroke MRI imaging protocol that included a non-contrast intracranial MRA using a 3D time-of-flight sequence, axial DWI, axial T2 GRE/SWI, and axial FLAIR sequences. Variability of practice among the on-call attending physicians lead to some patients additionally receiving gadolinium-enhanced extracranial MRA. The decision to obtain enhanced neck imaging was solely at the discretion of the stroke team on-call, based on their clinical judgement, and practice style.

### Statistical Analysis

Nominal variables were first summarized using frequency descriptive analysis. Continuous variables were summarized using mean, standard deviation, quartiles, and interquartile range. Normality of the variables was tested by the Shapiro-Wilk test. A univariate analysis was performed. Continuous variables were compared using a Mann-Whitney test, and nominal variables were compared using a Chi-square test. Linear models as well as a logistic regression model were performed, adjusting for initial NIHSS, transfer paradigm (study center vs. referral center), occlusion site, performing a diagnostic angiogram during MT, gender, stroke etiology, and IV thrombolysis. These variables were chosen by distribution examination and based on published literature. Non-normally distributed continuous variables were logarithmically transformed to be used in a linear regression. Adequacy of the regression model was assessed using QQ-plots and residual plots. *P*-values < 0.05 were considered statistically significant. All statistical analyses were performed using R Statistical Software (version 3.4.2).

## Results

From January 2012 to December 2017, 912 AIS patients with LVO underwent MT based on MRI pre-intervention screening. Gadolinium-enhanced extracranial MRA was performed in 288 patients (31.6%). Patients' characteristics and clinical outcomes are presented in Table 1. MT technical characteristics and time delays are presented in Table 2. Ninety-six percent of patients who did not receive gadolinium-enhanced extracranial imaging were referred patients and incurred significantly longer onset-groin puncture and onset to recanalization time delays. Of the referred patients, 79% (601/760) did not receive gadolinium-enhanced extracranial imaging vs. only 15% (23/152) of patients admitted directly at our comprehensive stroke center.

**Table 1:** Patients characteristics and clinical outcomes.

	**All *N* = 912 (100)**	**Patients with gadolinium-enhanced extracranial MRA *N* = 288 (32)**	**Patients without gadolinium-enhanced extracranial MRA *N* = 624 (68)**	***P*-value**
**Gender**				0.02
Female	435 (48)	121 (41.0)	314 (50.3)
Age (years)	67.5 ± 15.3	67.3 ± 15.0	67.6 ± 15.4	NS
Initial NIHSS	16 (10-20)	17 (11-21)	16 (10-20)	NS
**Transfer paradigm**				< 0.0001
Study center	152 (16.7)	129 (44.8)	23 (3.7)	
Referral center	760 (83.3)	159 (55.2)	601 (96.3)	
**Side**				NS
Right	402 (44.1)	120 (41.7)	282 (45.2)	
Left	448 (49.1)	144 (50.0)	304 (48.7)	
Posterior circulation	62 (6.8)	24 (8.3)	38 (6.1)	
**Occlusion site**				0.02
MCA-M1	436 (47.8)	115 (39.9)	321 (51.4)	
MCA- M2	122 (13.4)	41 (14.2)	81 (13.0)	
Terminal ICA	145 (15.9)	58 (20.1)	87 (13.9)	
Tandem (cervical ICA + intracranial)	147 (16.1)	50 (17.4)	97 (15.5)	
Basilar artery	62 (6.8)	24 (8.3)	38 (6.1)	
**Intravenous thrombolysis**				0.002
Yes	607 (66.6)	171 (59.4)	436 (69.9)	
No	305 (3.4)	117 (40.6)	188 (30.1)	
**Clinical outcome at 3 months**				
Good (mRS ≤ 2)	496 (54.4)	163 (56.6)	333 (53.4)	NS
Mortality	195 (21.4)	70 (24.3)	125 (20.0)	NS
**Stroke etiology**				NS
ICAD	220 (24.1)	72 (25)	148 (23.7)	
Cardio-embolic	418 (45.8)	136 (47.2)	282 (45.2)	
Dissection	42 (4.6)	17 (5.9)	25 (4.0)	
Other	232 (25.4)	63 (21.9)	169 (27.1)	

*Data presented as n (%), mean ± SD, or median (IQR). NS, not significant; ICAD, intracranial atherosclerotic disease*.

**Table 2:** MT characteristics.

	**All *N* = 912 (100)**	**Patients with gadolinium-enhanced extracranial MRA *N* = 288 (32)**	**Patients without gadolinium-enhanced extracranial MRA *N* = 624 (68)**	***P*-value**
**Time delays in min, median (IQR)**				
Onset-groin puncture	255 (209–310)	225 (178–305.0)	262 (220–315)	0.0001
Puncture-clot contact	25 (18–36)	24 (17–35)	26 (19–37)	NS
Puncture-recanalization	53 (34–82)	53 (33–83)	52 (34–82)	0.98
Onset-recanalization	320 (258–385)	289 (236–378)	330 (270–388)	0.0002
**Access failure**				NS
Yes	14 (1.5)	5 (1.7)	9 (1.4)	
No	898 (98.5)	283 (98.3)	615 (98.6)	
**Anesthesia**				NS
Conscious sedation	720 (79.0)	224 (77.8)	496 (79.5)	
General anesthesia	192 (21.0)	64 (22.2)	128 (20.5)	
**MT technique**				NS
Stent-retriever (SR)	286 (31.4)	93 (32.3)	193 (30.9)	
Contact aspiration (CA)	527 (57.8)	164 (56.9)	363 (58.2)	
Combined (SR+CA)	99 (10.9)	31 (10.8)	68 (10.9)	
**Cervical stenting/angioplasty**				NS
Yes	70 (7.7)	20 (6.9)	50 (8.0)	
No	842 (92.3)	268 (93.1)	574 (92.0)	
**Successful recanalization (TICI 2b or 3)**				NS
Yes	728 (79.8)	222 (77.1)	506 (81.1)	
No	184 (20.2)	66 (22.9)	118 (18.9)	
Procedural complications^*^				NS
Yes	151 (16.6)	49 (17.0)	102 (16.4)	
No	761 (83.4)	239 (83)	522 (83.7)	

*Data are presented as n (%), mean±SD, or median(IQR)*.

* *Intracranial embolus, dissection, perforation. NS, not significant*.

Groin puncture to clot contact or to recanalization time delays as well as rates of successful recanalization and procedural complications were similar between groups. All the potential cofounding variables presented in Tables 1, 2 were integrated in the multivariate analysis (results in Tables 3, 4). After adjusting for initial NIHSS, gender, transfer paradigm (study center vs. referral center), occlusion site, stroke etiology, and IV thrombolysis, the relative effect of performing gadolinium-enhanced extracranial MRA on groin puncture to clot contact time (RE = 0.93 [0.85–1.02], *p* = 0.14) and groin puncture to recanalization time (RE = 0.92 [0.83–1.03], *p* = 0.15) was not significant. Additionally, performing gadolinium-enhanced extracranial MRA was not associated with an increased rate of successful recanalization (OR = 0.93 [0.62–1.42], *p* = 0.74) or good clinical outcome (OR = 1.05 [0.73–1.52], *p* = 0.79) in the adjusted model.

**Table 3:** Results of multivariate analysis: relative effect of performing gadolinium-enhanced extracranial MRA on puncture-clot contact, puncture-recanalization, and procedural complications.

	**Relative effect with 95% confidence interval**	***P*-value univariate**	***P*-value multivariate**
Delay puncture-clot contact	0.93 [0.85–1.02]	0.15	0.14
Delay puncture-recanalization	0.92 [0.83–1.03]	0.98	0.15
Complications	0.81 [0.51–1.27]	0.88	0.36

**Table 4:** Results of multivariate analysis: Odds ratio for recanalization success and good clinical outcome among patients who underwent gadolinium-enhanced extracranial MRA prior to MT.

	**Odds-ratio with 95% confidence interval**	***P*-value univariate**	***P*-value multivariate**
Recanalization rate (TICI 2b-3)	0.93 [0.62–1.42]	0.19	0.74
Good clinical outcome at 3 months follow-up (mRS ≤ 2)	1.05 [0.73–1.52]	0.40	0.79

## Discussion

Our results show that performing gadolinium-enhanced extracranial MRA was not associated with delayed or shortened procedural time in our practice. Thrombectomy metrics (e.g., groin puncture to clot contact or to recanalization time) and outcomes (rates of successful recanalization and procedural complications) were similar and proportions of good clinical outcomes were also similar between patients who underwent extracranial MRA and those who did not.

Onset to groin puncture and onset to recanalization time were higher in the group who did not receive extracranial MRA, a finding we believe to be directly related to the observation that most patients in this group originated from a transfer center (96%) as groin puncture to recanalization time were similar between groups. A trend for referring centers not to perform extracranial MRA was highlighted in our analysis, as opposed to a more homogenous practice among our local practitioners at the study center who preferred to perform extracranial MRA almost systematically (84.8% of patients). We believe the absence of interventional providers and interventional management at the referring centers drives the local providers to omit the extracranial MRA for the sake of timely administrating intravenous thrombolysis (when indicated), shortening the door to transfer time and adhering to local departmental organization or to teleradiology recommendations.

Recent MT clinical trials have all included non-invasive intracranial documentation of LVO, an imaging screening paradigm applied at most practices. Obtaining concomitant extracranial vessel imaging might seem intuitive to providers in their quest for information that could add value to MT planning or performance. The most valuable information that extracranial imaging would provide to the operator include the type of aortic arch, presence of carotid stenosis, dissection or occlusion, and degree of vessel tortuosity. Extracranial imaging may also provide the medical clinician with valuable information pertaining to stroke etiology. Nevertheless, our study found no added benefit for gadolinium-enhanced extracranial MRA in MT patients during the acute phase compared with patients who received an unenhanced intracranial angiogram only. Although gadolinium-enhanced extracranial MRA may help in anticipating a difficult arch, no morphologic data based on extracranial MRA has been demonstrated beneficial for identifying AIS patients with a futile femoral access requiring a-priori radial or direct cervical puncture. Previous studies have identified morphologic factors that could interfere with cervical carotid stenting procedures (11, 12), but no such data is yet available for MT. On the other hand, as attractive as non-invasive extracranial MRA might be to some interventional and medical providers, it would never alter the indication for MT. In our practice, we would not withhold a thrombectomy attempt from an identified candidate based on morphological information obtained from extracranial MRA. The presence of a cervical dissection/stenosis is not a MT contra-indication; furthermore, the accuracy and reliability for the assessment of cervical internal carotid artery patency on non-invasive imaging is poor and hence insufficient for use in clinical practice (13). Lastly, the benefit of extracranial imaging in etiology assessment during the acute phase of stroke is yet to be demonstrated. Stroke etiology and risk factors stratification may be sought after in the subacute phase of treatment and the recent stroke guidelines do not imply or suggest otherwise (1).

Our study is limited by its retrospective observational design and sampling bias due to the single center data for MT. We did not record other time metrics (such as door-to-groin time) which could have been interesting to determine if the use of extracranial vascular imaging resulted in delay to treatment.A possible explanation for the absence of association found in our study could be that stroke physicians were able to select patients in whom extracranial vessel imaging was required, resulting in similar procedure times, and outcomes vs. patients who did not undergo extracranial vessel imaging. Randomized trials on this subject are warranted to draw firm conclusions.

## Conclusion

Our study showed that performing gadolinium-enhanced extracranial MRA in MT candidates was not associated with a modification of procedural time in our studied population. Further multi-centric studies or meta-analyses are necessary to confirm these findings and provide practice changing observations.

## Disclosure

The corresponding author (RF) has full access to all the data in the study and has final responsibility for the decision to submit for publication.

## Author Contributions

AG, NK, RB, MM, SS, HR, GC, J-PD, SE, KZ, PC, MH, NB-N, MB, MP, and RF contributed to the study design, data collection, and manuscript writing. KZ and PC performed statistical analyses.

### Conflict of Interest Statement

The authors declare that the research was conducted in the absence of any commercial or financial relationships that could be construed as a potential conflict of interest.
